# Clinical and genetic features of hereditary transthyretin amyloidosis with polyneuropathy in China: insights from case analysis and literature review

**DOI:** 10.3389/fgene.2026.1715134

**Published:** 2026-02-19

**Authors:** Xiaoyang Yuan, Yajun Lv, Xue Wang, Bing Han, Bingchuan Xie

**Affiliations:** 1 Department of Neurology, The First Hospital of Hebei Medical University, Shijiazhuang, Hebei, China; 2 Department of Neurology, Hebei Hospital of Xuanwu Hospital Capital Medical University, Shijiazhuang, Hebei, China; 3 Department of Minimally Invasive Spine Surgery, Hebei Hospital of Xuanwu Hospital Capital Medical University, Shijiazhuang, Hebei, China

**Keywords:** ATTRv-PN, hereditary transthyretin amyloidosis, polyneuropathy, sural nerve biopsy, TTR

## Abstract

**Background:**

Hereditary transthyretin amyloidosis with polyneuropathy (ATTRv-PN) is a progressive multisystem disorder caused by pathogenic *TTR* variants. Aim of this study is to delineate the clinical and genetic feature of ATTRv-PN in China and to evaluate diagnostic strategies for earlier and accurate recognition.

**Methods:**

We investigated two genetically confirmed Chinese patients with ATTRv-PN through comprehensive assessments, including electrophysiology, sural nerve biopsy, autonomic testing, cardiac imaging, neuroimaging, and cerebrospinal fluid analysis. Additionally, logistic regression was performed on 70 reported p.Val50Met cases to examine the relationship between age at onset and amyloid detection in sural nerve biopsies.

**Results:**

The two patients carried the p.Val50Met and p.Glu74Gly variants in the *TTR* gene, respectively. Both presented with distal paresthesia as the initial symptom and exhibited length-dependent, axonal-predominant sensorimotor polyneuropathy. Autonomic manifestations included alternating diarrhea/constipation and post-exertional hyperhidrosis. Subclinical cardiac abnormalities were identified, encompassing elevated biomarker of early cardiac strain, valvular regurgitation, left ventricular hypertrophy, and late gadolinium enhancement. In patient 1, sural nerve pathology showed marked loss of myelinated and unmyelinated fibers with perivascular lymphocytic infiltration but no amyloid deposition. Logistic regression revealed no significant association between age at onset and biopsy amyloid positivity (*p* = 0.91).

**Conclusion:**

This study expands the clinical and genetic landscape of ATTRv-PN in China and highlighted the heterogeneity of amyloid detection in nerve biopsies. Accurate and timely diagnosis requires an integrated approach combining clinical, electrophysiological, pathological, genetic, and multimodal imaging assessments to facilitate early initiation of disease-modifying therapies.

## 1 Introduction

Hereditary transthyretin amyloidosis (ATTRv) is a progressive, multisystem disorder caused by pathogenic variants in the transthyretin (*TTR)* gene. Mutant TTR destabilizes the transthyretin tetramer, leading to misfolding, amyloid fibril formation, and deposition in the peripheral nerves, heart, and other organs (Adams et al., 2021). Among the spectrum of clinical manifestations, polyneuropathy (ATTRv-PN) is the most common and often the initial presentation, typically manifesting as length-dependent sensory-motor neuropathy accompanied by autonomic dysfunction. To date, more than 140 pathogenic variants have been identified in this small, 147-amino-acid protein (NM_000371.4; http://amyloidosismutations.com/mut-attr.php), with p.Val50Met (historically p.Val30Met) representing the most prevalent. This variant was first reported in endemic regions of Sweden, Portugal, and Japan (Holmgren et al., 1988; Ikeda et al., 2002; Sousa et al., 1995), but has since been recognized worldwide.

Despite its autosomal dominant inheritance, ATTRv exhibits striking incomplete penetrance and marked heterogeneity in age at onset, complicating diagnosis and obscuring true disease prevalence. In early-onset p.Val50Met carriers, penetrance rises from 10% by age 40%–71% by age 90 in Swedish cohorts (Gorram et al., 2021). Late-onset phenotypes pose further diagnostic challenges, often resulting in delayed recognition. The interplay among carrier frequency, age at onset, and penetrance profoundly shapes the epidemiology of ATTRv, underscoring the importance of population-specific investigations.

Unlike the extensive investigations conducted in Japan, studies of ATTRv in China remain relatively limited. Here, we report two Chinese patients with genetically confirmed ATTRv-PN, providing detailed clinical and pathological characterization. By reviewing previously published cases with p.Val50Met, we explore the potential relationship between age at onset and the detectability of amyloid deposits, and we emphasize an integrated diagnostic strategy that combines clinical, genetic, and pathological data to enable earlier recognition in underdiagnosed populations.

## 2 Materials and methods

### 2.1 Genetic study

Genomic DNA was analyzed through PCR amplification and Sanger sequencing (Kangxu Medical Laboratory, Beijing, China) for all coding exons of the *TTR* gene.

### 2.2 Sural nerve pathology

A sural nerve biopsy was performed on patient 1, and portions of the specimen were fixed in 10% neutral-buffered formalin, paraffin-embedded, and sectioned for routine hematoxylin and eosin (H&E) staining. Congo red staining with polarized light microscopy was performed to detect amyloid deposits. Semithin sections prepared from resin-embedded tissue were stained with methylene blue to assess myelinated fiber density and morphology. Immunohistochemistry was conducted using antibodies against myelin basic protein (MBP, monoclonal) and neurofilament (NF, monoclonal). A polymer-based horseradish peroxidase system with diaminobenzidine (DAB) was used for visualization.

For ultrastructural evaluation, separate nerve tissue was fixed in 2.5% glutaraldehyde, post-fixed in 1% osmium tetroxide, and embedded in EPON resin. Ultrathin sections were stained with uranyl acetate and lead citrate and examined under a transmission electron microscope to assess axonal and myelin ultrastructure and amyloid fibrils.

### 2.3 Literature review of amyloid deposition in sural nerve of patients with the p.Val50Met variant

A systematic search of the PubMed database was performed to identify published English-language reports describing sural nerve biopsies in patients with FAP or TTR amyloidosis. Search terms included “familial amyloid polyneuropathy” OR “transthyretin amyloidosis” in combination with “sural nerve.” We included cases in which patients carried the p.Val50Met (formerly p.Val30Met) *TTR* variant and for which individual clinical and histopathological data were available. To assess whether age at onset predicted amyloid deposition in sural nerve biopsies, we performed a logistic regression analysis using RStudio (Build 496).

## 3 Results

### 3.1 Clinical summary

#### 3.1.1 Patient 1

A 70-year-old male with no family history presented with a nine-month history of progressive sensory disturbances, beginning with numbness, coldness, and tingling paresthesia in the hands and distal lower extremities. Over time, he developed ascending stiffness from the ankles to the knees, followed by worsening gait impairment and alternating diarrhea and constipation. Neurological examination showed diminished pain sensation in the distal upper and lower extremities. Motor strength was preserved, and no cerebellar or cranial nerve abnormalities were noted. Cardiopulmonary and abdominal examinations were unremarkable.

Laboratory studies revealed elevated albumin level (58.8 g/L; normal range: 40∼55 g/L) and N-terminal pro-B-type natriuretic peptide (NT-proBNP, 530 pg/mL; normal range: <125 pg/mL), with otherwise unremarkable cardiac biomarkers (troponin I and myoglobin), serum immunoglobulins, urine protein electrophoresis, and cerebrospinal fluid (CSF) analysis. He had no history of diabetes mellitus. Echocardiography demonstrated mitral and tricuspid regurgitation, while abdominal ultrasound revealed mild hepatic parenchymal heterogeneity. Autonomic testing identified impaired vagal and adrenergic cardiovascular responses. Brain MRI showed lacunar infarcts in the bilateral basal ganglia and ischemic white matter lesions. Additionally, vascular studies showed diffuse intima-media thickening with bilateral atherosclerotic plaques and an intramuscular venous thrombosis in the left calf. Electrophysiological studies confirmed a length-dependent peripheral neuropathy with mixed axonal and demyelinating features, predominantly axonal.

#### 3.1.2 Patient 2

**FIGURE 1:**
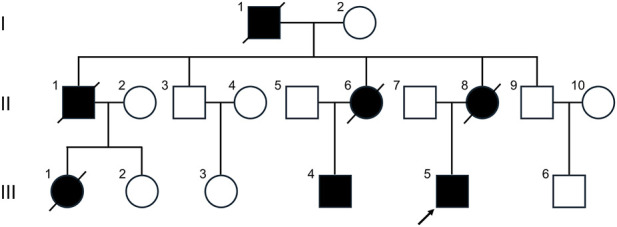
Family pedigree of patient 2. The arrow (↑) indicates the proband.

A 31-year-old man (III-5) with a strong family history of ATTRv (grandfather, mother, aunt, uncle, and cousins) presented with progressive liver dysfunction following liver transplantation at age 29 (Figure 1). Six months prior to evaluation, he developed lower limb paresthesia, including numbness and activity-induced neuropathic pain, accompanied exercise-related weakness and post-exertional hyperhidrosis. Symptoms progressed without fever, cardiac complaints, or gastrointestinal disturbances. Neurological examination revealed preserved muscle strength, absent deep tendon reflexes in the lower extremities, and reduced pain sensation along the lateral and anterior aspects of both legs. Reflexes and strength in the upper limbs were normal. Cardiovascular, pulmonary, and abdominal examinations were unremarkable.

CSF analysis demonstrated markedly elevated protein (1,058.2 ng/L; normal range: 150.0∼450.0 ng/L) with otherwise normal parameters. Cardiac evaluation revealed concentric left ventricular hypertrophy with diastolic dysfunction on echocardiography. Cardiac MRI confirmed diffuse ventricular wall thickening, most prominent in basal and mid segments, with late gadolinium enhancement involving both ventricles and the right atrium. Abdominal ultrasonography, brain MRI, and ophthalmoscopic examination were normal.

Electrophysiological studies evolved over time: nerve conduction studies (NCS) at age 23 were normal, but at age 31 they revealed absent sensory potentials, reduced motor conduction velocities (MCV) in median nerves (35∼48 m/s) and ulnar nerves (40∼52 m/s), as well as diminished compound muscle action potentials (CMAP, 1.2∼1.8 mV). Tibial motor responses were unelicitable, consistent with a predominantly axonal sensorimotor neuropathy with secondary demyelinating features.

### 3.2 Genetic findings

In Patient 1, Sanger sequencing revealed a heterozygous missense variant in *TTR* exon 2, c.148G>A (p.Val50Met; NM_000371.4). This variant is present in the gnomAD database (v4.1.0; accessed June 2025) with an allele frequency of 92/1,614,098 overall and 2/44,886 among East Asian individuals.

In Patient 2, a heterozygous missense variant in *TTR* exon 3, c.221A>G (p.Glu74Gly; NM_000371.4), was identified. This variant is absent from population databases and segregates with disease in his affected cousins (III-1 and III-4), supporting its pathogenicity (Figure 1).

### 3.3 Sural nerve pathological findings (patient 1)

**FIGURE 2:**
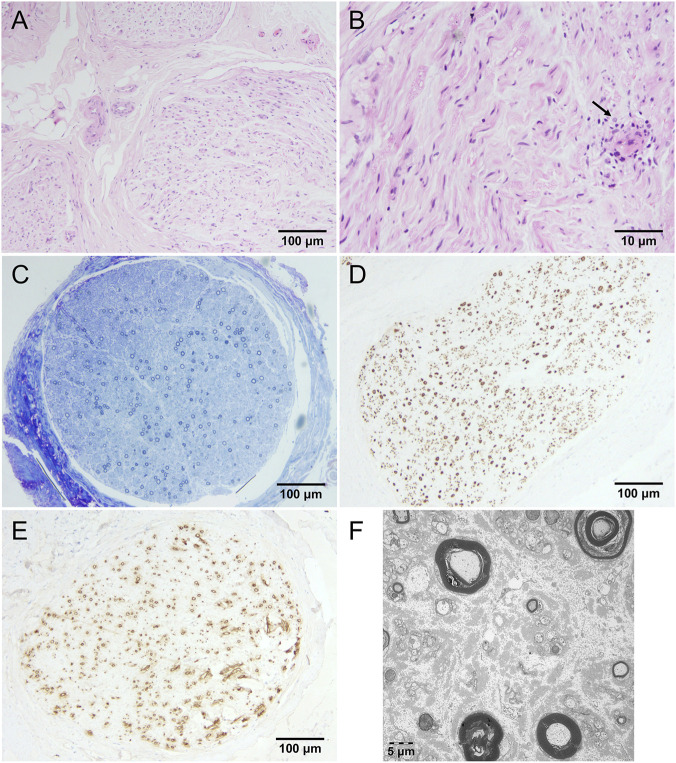
Sural nerve pathology of patient 1. **(A)** H&E staining showing multiple nerve fascicles and small blood vessels within interfascicular connective tissue. **(B)** H&E staining highlighting perivascular lymphocytic infiltration surrounding an intrafascicular small vessel (black arrow). **(C)** Toluidine blue staining showing moderate loss of both large and small myelinated fibers. **(D,E)** Immunohistochemistry of myelin basic protein (MBP) and neurofilament (NF) revealing substantial reduction of myelinated fibers. **(F)** Electron microscopy illustrating loss of myelinated and unmyelinated fibers, focal thickening and lamellation of myelin sheaths, and axonal degeneration.

Histopathological analysis of the sural nerve revealed sparse perivascular lymphocytic infiltration around a single small vessel, without definitive evidence of vasculitis (H&E staining; Figures 2A,B). Semithin toluidine blue staining demonstrated a moderate reduction of both large and small myelinated fibers, with no myelin ovoids or onion bulb formations (Figure 2C). Congo red staining was negative for amyloid deposition (data not shown).

Immunohistochemistry for MBP and NF confirmed loss of myelinated fibers (Figures 2D,E). Electron microscopy (EM) revealed a marked reduction in both myelinated and unmyelinated fibers, focal thickening and lamellation of myelin sheaths, and mild axonal degeneration in a subset of fibers, without evidence of amyloid fibrils (Figure 2F).

### 3.4 Literature review of described patients with the p.Val50Met variant

We collected 70 patients with the p.Val50Met variant for whom individual age at onset and sural nerve biopsy histopathological data were available (Bekircan-Kurt et al., 2015; Cappellari et al., 2011; Du et al., 2021; Koike et al., 2011; Koike et al., 2004; Koike and Sobue, 2012; Kollmer et al., 2017; Mathis et al., 2012; Meng et al., 2015; Yoshioka et al., 2001). The cohort included 58 males and 12 females. Congo red staining was used in all cases, with or without adjunctive TTR immunohistochemistry. Amyloid deposits were detected in 51 patients. Based on age at symptom onset, 15 patients were classified as early-onset (<50 years) and 55 as late-onset (≥50 years) (Supplementary Table S1).

**FIGURE 3:**
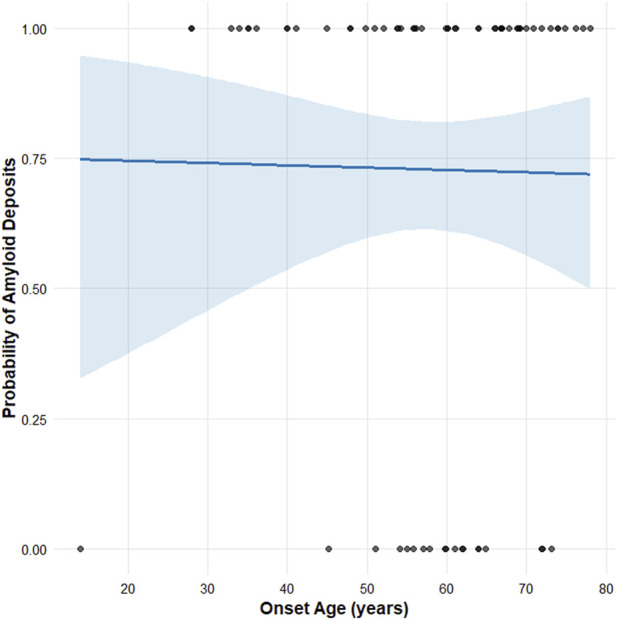
Logistic regression analysis of 70 reported p.Val50Met cases with available data on age at onset and amyloid detection with sural nerve biopsy. No significant association was observed between age at onset and biopsy amyloid positivity (β = −0.0023, standard error = 0.020, *p* = 0.91).

Logistic regression analysis revealed no significant association between onset age and amyloid detection (β = −0.0023, standard error = 0.020, *p* = 0.91), indicating that the likelihood of amyloid deposition was not dependent on the age at symptom onset (Figure 3).

## 4 Discussion

ATTRv-PN remains challenging to distinguish from other axonal or demyelinating neuropathies due to its heterogenous presentation. In this study, we report two Chinese patients who both present with paresthesia as the initial symptom. Electrophysiological studies demonstrated length-dependent, axonal-predominant sensorimotor polyneuropathy, consistent with typical ATTRv-PN. Genetic analysis identified pathogenic *TTR* variants in both patients: p.Val50Met in patient 1, the most prevalent variant globally, including in China, and p.Glu74Gly in patient 2, the third most frequently detected *TTR* variant in the Chinese population (Chu et al., 2025).

Autonomic dysfunction is a common and often disabling feature of ATTRv-PN. In patient 1, clinical manifestations included alternating diarrhea and constipation, and autonomic testing demonstrated impaired vagal and adrenergic cardiovascular responses, consistent with autonomic neuropathy (Barroso et al., 2022). Patient 2 experienced post-exertional hyperhidrosis, illustrating the variability of autonomic manifestations. Early recognition and proactive management of autonomic symptoms are essential to improve quality of life and prognosis in ATTRv-PN.

Beyond neuropathy, ATTR frequently involves the heart, with cardiomyopathy (ATTRv-CM) representing the leading cause of morbidity and mortality. Notably, neither of our two patients reported cardiac symptoms. Nevertheless, laboratory and imaging assessments revealed subclinical cardiac involvement. In patient 1, NT-proBNP was modestly elevated (530 pg/mL), suggesting early cardiac strain, a sensitive marker of myocardial involvement in systemic amyloidosis even in the absence of troponin or myoglobin elevation (Perfetto et al., 2022). Echocardiography further demonstrated mitral and tricuspid regurgitation, potentially reflecting amyloid infiltration of the valvular apparatus or secondary hemodynamic alterations. Patient 2 exhibited more pronounced involvement, with concentric left ventricular hypertrophy and diastolic dysfunction on echocardiography, confirmed by cardiac MRI showing diffuse ventricular thickening and late gadolinium enhancement of both ventricles and the right atrium, hallmarks of amyloid cardiomyopathy (Mohseni et al., 2025; Scheel et al., 2022).

However, ATTR-specific cardiac confirmation using ^99m^Technetium-pyrophosphate (^99m^Tc-PYP) scintigraphy or endomyocardial biopsy was not performed, which limits definitive etiological attribution of the observed cardiac findings. Given evidence from recent family screening studies demonstrating that PYP scintigraphy can identify ATTRv-CM even in the absence of left ventricular hypertrophy (Muller et al., 2024), the extent of ATTR-specific myocardial deposition may have been underestimated. These observations nonetheless highlight that cardiac involvement in ATTRv-PN may be clinically silent yet detectable by sensitive biomarkers and multimodal imaging reinforcing the need for systematic amyloid-specific cardiovascular evaluation.

Vascular involvement may also complicate ATTRv-PN. Neuroimaging of patient 1 revealed lacunar infarcts in the basal ganglia and ischemic white matter lesions, consistent with small-vessel cerebrovascular disease. Although ATTRv is not classically linked to ischemic stroke, these findings may reflect amyloid-related vasculopathy or coexisting vascular risk factors. Vascular ultrasound further demonstrated diffuse intima-media thickening and bilateral atherosclerotic plaques, indicative of systemic vasculopathy, and the presence of intramuscular venous thrombosis in the calf further highlights the vascular vulnerability. In patient 2, the markedly elevated CSF protein (1,058.2 ng/L) with otherwise normal parameters suggests a disruption of the blood-CSF barrier, a finding that could be misinterpreted as chronic inflammatory demyelinating polyneuropathy (CIDP), but is increasingly recognized in ATTRv (Chao et al., 2025).

In patient 1, who carried the p.Val50Met variant, sural nerve biopsy did not reveal amyloid deposition on Congo red staining or electron microscopy. The sensitivity of amyloid detection in nerve tissue varies considerably across cohorts. In Japanese series of p.Val50Met ATTRv-PN, amyloid deposits were detected in 18/20 late-onset patients (Misu et al., 1999) and 15/19 mixed-onset cases (Koike et al., 2004), with frequencies comparable to European reports, including 87.5% in Italy (21/24) and 86% in Portugal (Fernandes et al., 2019; Luigetti et al., 2020). By contrast, in a Chinese series, Congo red positivity was observed in only 40% of sural nerve samples overall, with higher rates in early-onset (55.6%) compared with late-onset cases (27.3%); among patients with p.Val50Met, only 30% (3/10; 1 early-onset, 9 late-onset) were positive (Du et al., 2021). Taken together, these data suggest that age at onset may affect the likelihood of detecting amyloid deposits in nerve tissue.

We therefore reviewed reported p.Val50Met patients with available data on both age at onset and sural nerve amyloid deposition, and performed a logistic regression analysis, including 15 early-onset and 55 late-onset cases. The analysis showed no significant association between age at symptom onset and amyloid detection (*p* = 0.91). However, because this analysis was based on published literature data, it is subject to inherent limitations, including publication bias and heterogeneity in study design, reporting practices, and biopsy methodologies, and should therefore be interpreted with caution.

Notably, early- and late-onset ATTRv-PN differ in amyloid fibril composition, with early-onset cases exhibiting thick, full-length TTR fibrils and strong Congo red affinity, whereas late-onset cases often harbor thinner, fragmented fibrils with weaker congophilia, which may reduce biopsy sensitivity (Ihse et al., 2008; Koike et al., 2018). Additional factors, including variability in staining procedures, inter-observer interpretation, and the patchy distribution of amyloid deposits, may also contribute to false-negative results. Collectively, these findings highlight the limitations of sural nerve biopsy as a diagnostic tool and underscore the need for larger cohorts and standardized detection protocols to better clarify the relationship between disease onset age, fibril characteristics, and amyloid deposition rates.

In patient 1, sural nerve biopsy revealed perivascular lymphocytic infiltration around a small vessel, a finding that, although uncommon, has been reported in other ATTRv-PN cases. Similar inflammatory changes have been observed in sural nerves from multiple patients, indicating that lymphocytic infiltration can be part of the pathological spectrum of ATTRv-PN (Luigetti et al., 2020). Beyond local nerve pathology, systemic immune alterations have also been described, including dysregulation of circulating cytokines such as interferon (IFN)-γ, IFN-α, IL-6, IL-7, and IL-33 during disease progression (Plantone et al., 2023). Collectively, these findings suggest that, while ATTRv-PN is classically regarded as a non-inflammatory neuropathy, neuroinflammatory mechanisms may contribute to disease heterogeneity and progression, with potential implications for diagnosis and therapeutic strategies.

Current therapeutic strategies for ATTRv aim to prevent amyloid formation either by stabilizing the TTR tetramer or by reducing its production. Tafamidis, the first approved disease-modifying therapy for both ATTRv-PN and ATTRv-CM, selectively binds to the T4 site of TTR, stabilizing the tetramer and slows disease progression, with the greatest efficacy when initiated early (Carroll et al., 2022). In patients with ATTRv-CM, tafamidis significantly reduced mortality and cardiovascular hospitalizations, particularly in those treated at early stages of cardiac involvement (Maurer et al., 2018). Gene-silencing therapies, including the antisense oligonucleotide (inotersen) and the small interfering RNA (patisiran), suppress hepatic TTR production and have demonstrated clinical benefit in slowing neurological progression (Adams et al., 2018; Benson et al., 2018; Coelho et al., 2020). More recently, *in vivo* CRISPR-Cas9 gene editing has emerged as a potentially curative approach. NTLA-2001, delivered via lipid nanoparticles, induces permanent *TTR* gene disruption, achieving up to 95% reduction in serum TTR levels after a single infusion with an acceptable safety profile (Gillmore et al., 2021).

The efficacy of these treatments for ATTRv is strongly dependent to disease stage, underscoring the critical importance of early diagnosis and longitudinal monitoring in asymptomatic carriers. The Transthyretin Amyloidosis Outcomes Survey (THAOS), a global observational study, reported that more than one-third of asymptomatic *TTR* variant carriers developed symptomatic ATTRv amyloidosis within a median of 2.2 years after enrollment (Coelho et al., 2022). Supporting these findings, a longitudinal study of 98 presumed asymptomatic carriers with normal baseline NCS found that 11% exhibited NCS deterioration over a median follow-up of 5 years (Schulz et al., 2025). Although nerve biopsy may be required to confirm neuropathic involvement, it is not essential for initiating treatment in patients with red-flag symptoms or in clinically typical cases, such as those described in the present study. When histopathological evaluation is indicated, skin biopsy can provide information comparable to nerve biopsy while carrying a lower risk of complications.

In ATTRv-PN, small fiber neuropathy often precedes large fiber involvement, reflecting the early susceptibility of unmyelinated and thinly myelinated fibers to amyloid toxicity (Koike et al., 2004). Given this early neuropathic pattern, systematic assessment of small fibers using techniques such as skin biopsy, quantitative sensory testing, and corneal confocal microscopy is essential for detecting pathological changes. In parallel, the development and validation of pre-symptomatic biomarkers, like the circulating TTR monomers and serum glial fibrillary acidic protein (sGFAP), offering potential avenues identify early disease and guide timely therapeutic intervention (Costa-Rodrigues et al., 2024; Plantone et al., 2025).

## 5 Conclusion

The early and accurate diagnosis of ATTRv relies on a comprehensive, integrated approach encompassing clinical assessment, laboratory evaluation, genetic analysis, and pathological confirmation. Genetic testing of the *TTR* gene remains the standard for identifying pathogenic variants and at-risk family members. In ATTRv-PN patients, nerve biopsy demonstrating amyloid deposits provides definitive pathological evidence, although sensitivity may vary with disease stage, ancestry, and specific *TTR* variant. By periodically integrating these complementary diagnostic strategies, clinicians can accurately determine disease stage, enabling the timely initiation of disease-modifying therapies when they are most effective ultimately improving patient outcomes.

## Data Availability

The datasets presented in this article are not readily available because of ethical and privacy restrictions. Requests to access the datasets should be directed to the corresponding author.

## References

[B1] Adams D. Gonzalez-Duarte A. O'Riordan W. D. Yang C. C. Ueda M. Kristen A. V. (2018). Patisiran, an RNAi therapeutic, for hereditary transthyretin amyloidosis. N. Engl. J. Med. 379, 11–21. 10.1056/NEJMoa1716153 29972753

[B2] Adams D. Ando Y. Beirao J. M. Coelho T. Gertz M. A. Gillmore J. D. (2021). Expert consensus recommendations to improve diagnosis of ATTR amyloidosis with polyneuropathy. J. Neurol. 268, 2109–2122. 10.1007/s00415-019-09688-0 31907599 PMC8179912

[B3] Barroso F. A. Coelho T. Dispenzieri A. Conceicao I. Waddington-Cruz M. Wixner J. (2022). Characteristics of patients with autonomic dysfunction in the transthyretin amyloidosis outcomes survey (THAOS). Amyloid 29, 175–183. 10.1080/13506129.2022.2043270 35451899

[B4] Bekircan-Kurt C. E. Gunes N. Yilmaz A. Erdem-Ozdamar S. Tan E. (2015). Three Turkish families with different transthyretin mutations. Neuromuscul. Disord. 25, 686–692. 10.1016/j.nmd.2015.05.010 26115788

[B5] Benson M. D. Waddington-Cruz M. Berk J. L. Polydefkis M. Dyck P. J. Wang A. K. (2018). Inotersen treatment for patients with hereditary transthyretin amyloidosis. N. Engl. J. Med. 379, 22–31. 10.1056/NEJMoa1716793 29972757 PMC12611561

[B6] Cappellari M. Cavallaro T. Ferrarini M. Cabrini I. Taioli F. Ferrari S. (2011). Variable presentations of TTR-related familial amyloid polyneuropathy in seventeen patients. J. Peripher Nerv. Syst. 16, 119–129. 10.1111/j.1529-8027.2011.00331.x 21692911

[B7] Carroll A. Dyck P. J. de Carvalho M. Kennerson M. Reilly M. M. Kiernan M. C. (2022). Novel approaches to diagnosis and management of hereditary transthyretin amyloidosis. J. Neurol. Neurosurg. Psychiatry 93, 668–678. 10.1136/jnnp-2021-327909 35256455 PMC9148983

[B8] Chao C. C. Yang W. K. Yeh T. Y. Kan Y. Y. Wang Y. S. Lee K. J. (2025). Transthyretin variants impact blood-nerve barrier and neuroinflammation in amyloidotic neuropathy. Brain 148, 2537–2550. 10.1093/brain/awaf028 39874259

[B9] Chu X. Kang J. Xu J. Jiang H. Wu Z. Y. Wang Q. (2025). A multicenter study of hereditary transthyretin amyloidosis in China. Ann. Neurol. 97, 1158–1167. 10.1002/ana.27203 39976297

[B10] Coelho T. Yarlas A. Waddington-Cruz M. White M. K. Sikora Kessler A. Lovley A. (2020). Inotersen preserves or improves quality of life in hereditary transthyretin amyloidosis. J. Neurol. 267, 1070–1079. 10.1007/s00415-019-09671-9 31853709 PMC7109169

[B11] Coelho T. Conceicao I. Waddington-Cruz M. Keohane D. Sultan M. B. Chapman D. (2022). A natural history analysis of asymptomatic TTR gene carriers as they develop symptomatic transthyretin amyloidosis in the transthyretin amyloidosis outcomes survey (THAOS). Amyloid 29, 228–236. 10.1080/13506129.2022.2070470 35730447

[B12] Costa-Rodrigues D. Leite J. P. Saraiva M. J. Almeida M. R. Gales L. (2024). Transthyretin monomers: a new plasma biomarker for pre-symptomatic transthyretin-related amyloidosis. Amyloid 31, 202–208. 10.1080/13506129.2024.2368860 38946492

[B13] Du K. Li F. Wang H. Miao Y. Lv H. Zhang W. (2021). Hereditary transthyretin amyloidosis in mainland China: a unicentric retrospective study. Ann. Clin. Transl. Neurol. 8, 831–841. 10.1002/acn3.51328 33739616 PMC8045954

[B14] Fernandes A. Coelho T. Rodrigues A. Felgueiras H. Oliveira P. Guimaraes A. (2019). Clinicopathological correlations of sural nerve biopsies in TTR Val30Met familial amyloid polyneuropathy. Brain Commun. 1, fcz032. 10.1093/braincomms/fcz032 32954271 PMC7425381

[B15] Gillmore J. D. Gane E. Taubel J. Kao J. Fontana M. Maitland M. L. (2021). CRISPR-Cas9 *in vivo* gene editing for transthyretin amyloidosis. N. Engl. J. Med. 385, 493–502. 10.1056/NEJMoa2107454 34215024

[B16] Gorram F. Olsson M. Alarcon F. Nuel G. Anan I. Plante-Bordeneuve V. (2021). New data on the genetic profile and penetrance of hereditary Val30Met transthyretin amyloidosis in Sweden. Amyloid 28, 84–90. 10.1080/13506129.2020.1841623 33146042

[B17] Holmgren G. Holmberg E. Lindstrom A. Lindstrom E. Nordenson I. Sandgren O. (1988). Diagnosis of familial amyloidotic polyneuropathy in Sweden by RFLP analysis. Clin. Genet. 33, 176–180. 10.1111/j.1399-0004.1988.tb03434.x 2896079

[B18] Ihse E. Ybo A. Suhr O. Lindqvist P. Backman C. Westermark P. (2008). Amyloid fibril composition is related to the phenotype of hereditary transthyretin V30M amyloidosis. J. Pathol. 216, 253–261. 10.1002/path.2411 18729067

[B19] Ikeda S. Nakazato M. Ando Y. Sobue G. (2002). Familial transthyretin-type amyloid polyneuropathy in Japan: clinical and genetic heterogeneity. Neurology 58 (58), 1001–1007. 10.1212/wnl.58.7.1001 11940682

[B20] Koike H. Sobue G. (2012). Late-onset familial amyloid polyneuropathy in Japan. Amyloid 19 (Suppl. 1), 55–57. 10.3109/13506129.2012.674580 22506939

[B21] Koike H. Misu K. Sugiura M. Iijima M. Mori K. Yamamoto M. (2004). Pathology of early-vs late-onset TTR Met30 familial amyloid polyneuropathy. Neurology 63, 129–138. 10.1212/01.wnl.0000132966.36437.12 15249622

[B22] Koike H. Hashimoto R. Tomita M. Kawagashira Y. Iijima M. Tanaka F. (2011). Diagnosis of sporadic transthyretin Val30Met familial amyloid polyneuropathy: a practical analysis. Amyloid 18, 53–62. 10.3109/13506129.2011.565524 21463231

[B23] Koike H. Nishi R. Ikeda S. Kawagashira Y. Iijima M. Sakurai T. (2018). The morphology of amyloid fibrils and their impact on tissue damage in hereditary transthyretin amyloidosis: an ultrastructural study. J. Neurol. Sci. 394, 99–106. 10.1016/j.jns.2018.09.011 30243104

[B24] Kollmer J. Sahm F. Hegenbart U. Purrucker J. C. Kimmich C. Schonland S. O. (2017). Sural nerve injury in familial amyloid polyneuropathy: MR neurography vs clinicopathologic tools. Neurology 89, 475–484. 10.1212/WNL.0000000000004178 28679600

[B25] Luigetti M. Romozzi M. Bisogni G. Cardellini D. Cavallaro T. Di Paolantonio A. (2020). hATTR pathology: nerve biopsy results from Italian referral centers. Brain Sci. 10, 10. 10.3390/brainsci10110780 33114611 PMC7692609

[B26] Mathis S. Magy L. Diallo L. Boukhris S. Vallat J. M. (2012). Amyloid neuropathy mimicking chronic inflammatory demyelinating polyneuropathy. Muscle Nerve 45, 26–31. 10.1002/mus.22229 22190302

[B27] Maurer M. S. Schwartz J. H. Gundapaneni B. Elliott P. M. Merlini G. Waddington-Cruz M. (2018). Tafamidis treatment for patients with transthyretin amyloid cardiomyopathy. N. Engl. J. Med. 379, 1007–1016. 10.1056/NEJMoa1805689 30145929

[B28] Meng L. C. Lyu H. Zhang W. Liu J. Wang Z. X. Yuan Y. (2015). Hereditary transthyretin amyloidosis in eight Chinese families. Chin. Med. J. Engl. 128, 2902–2905. 10.4103/0366-6999.168048 26521788 PMC4756886

[B29] Misu K. Hattori N. Nagamatsu M. Ikeda S. Ando Y. Nakazato M. (1999). Late-onset familial amyloid polyneuropathy type I (transthyretin Met30-associated familial amyloid polyneuropathy) unrelated to endemic focus in Japan. Clinicopathological and genetic features. Brain 122 (10), 1951–1962. 10.1093/brain/122.10.1951 10506096

[B30] Mohseni A. Zandieh G. Porter K. Pozzessere C. Wagle A. Borhani A. (2025). Cardiac amyloidosis: a comprehensive review of imaging findings. Acad. Radiol. 32, 2517–2528. 10.1016/j.acra.2025.01.022 39893144

[B31] Muller S. A. Peiró-Aventin B. Biagioni G. Tini G. Saturi G. Kronberger C. (2024). Evaluation of the 2021 ESC recommendations for family screening in hereditary transthyretin cardiac amyloidosis. Eur. J. Heart Fail 26, 2025–2034. 10.1002/ejhf.3339 38887861

[B32] Perfetto F. Zampieri M. Fumagalli C. Allinovi M. Cappelli F. (2022). Circulating biomarkers in diagnosis and management of cardiac amyloidosis: a review for internist. Intern Emerg. Med. 17, 957–969. 10.1007/s11739-022-02958-2 35325395 PMC9135845

[B33] Plantone D. Primiano G. Righi D. Romano A. Luigetti M. De Stefano N. (2023). Current evidence supporting the role of immune response in ATTRv amyloidosis. Cells 12, 12. 10.3390/cells12192383 37830598 PMC10572348

[B34] Plantone D. Luigetti M. Manco C. Romano A. Leonardi L. Guglielmino V. (2025). Elevated serum concentrations of GFAP in hereditary transthyretin amyloidosis since pre-symptomatic stages. J. Neurol. 272, 340. 10.1007/s00415-025-13072-6 40232501 PMC12000116

[B35] Scheel P. J. 3rd Mukherjee M. Hays A. G. Vaishnav J. (2022). Multimodality imaging in the evaluation and prognostication of cardiac amyloidosis. Front. Cardiovasc Med. 9, 787618. 10.3389/fcvm.2022.787618 35402557 PMC8989413

[B36] Schulz N. Beauvais D. Cauquil C. Labeyrie C. Iliescu I. Francou B. (2025). Intracutaneous amyloid deposition is associated with nerve conduction studies deterioration in presumed asymptomatic pathogenic variant TTR carriers. Eur. J. Neurol. 32, e70277. 10.1111/ene.70277 40653952 PMC12256769

[B37] Sousa A. Coelho T. Barros J. Sequeiros J. (1995). Genetic epidemiology of familial amyloidotic polyneuropathy (FAP)-type I in povoa do varzim and vila do conde (north of Portugal). Am. J. Med. Genet. 60, 512–521. 10.1002/ajmg.1320600606 8825887

[B38] Yoshioka A. Yamaya Y. Saiki S. Hirose G. Shimazaki K. Nakamura M. (2001). A case of familial amyloid polyneuropathy homozygous for the transthyretin Val30Met gene with motor-dominant sensorimotor polyneuropathy and unusual sural nerve pathological findings. Arch. Neurol. 58, 1914–1918. 10.1001/archneur.58.11.1914 11709003

