# Supplementary material for: Clinical and genetic features of hereditary transthyretin amyloidosis with polyneuropathy in China: insights from case analysis and literature review
Source: Front Genet. 2026 Feb 19;17:1715134. doi: 10.3389/fgene.2026.1715134 (PMC12960095; doi:10.3389/fgene.2026.1715134)
Supplement: Supplementary file 1 [file DataSheet1.pdf]

Supplementary Table 1 Summary of 70 reported ATTRv-PN p.Val50Met cases with age at onset and amyloid detection in sural nerve biopsies

Case	Gender	Onset	Amyloid deposit	Reference
1	M	45	Yes	Yoshioka A, et al. 2001.
2	M	35	Yes	Koike H, et al. 2004.
3	F	33	Yes	Koike H, et al. 2004.
4	M	35	Yes	Koike H, et al. 2004.
5	M	36	Yes	Koike H, et al. 2004.
6	M	40	Yes	Koike H, et al. 2004.
7	M	28	Yes	Koike H, et al. 2004.
8	F	34	Yes	Koike H, et al. 2004.
9	F	41	Yes	Koike H, et al. 2004.
10	M	64	Yes	Koike H, et al. 2004.
11	M	52	Yes	Koike H, et al. 2004.
12	M	67	Yes	Koike H, et al. 2004.
13	M	77	Yes	Koike H, et al. 2004.
14	M	56	Yes	Koike H, et al. 2004.
15	M	56	Yes	Koike H, et al. 2004.
16	M	61	Yes	Koike H, et al. 2004.
17	M	64	No	Cappellari M, et al. 2011.
18	M	60	Yes	Cappellari M, et al. 2011.
19	M	78	Yes	Cappellari M, et al. 2011.
20	M	60	Yes	Cappellari M, et al. 2011.
21	M	72	Yes	Cappellari M, et al. 2011.
22	M	67	Yes	Cappellari M, et al. 2011.
23	F	74	Yes	Cappellari M, et al. 2011.
24	M	64	No	Cappellari M, et al. 2011.
25	M	55	No	Cappellari M, et al. 2011.
26	M	72	No	Cappellari M, et al. 2011.
27	F	45	No	Cappellari M, et al. 2011.
28	M	62	No	Cappellari M, et al. 2011.
29	M	14	No	Cappellari M, et al. 2011.
30	M	60	No	Cappellari M, et al. 2011.
31	M	57	No	Cappellari M, et al. 2011.
32	F	56	No	Cappellari M, et al. 2011.
33	M	51	No	Cappellari M, et al. 2011.
34	M	66	Yes	Mathis S, et al. 2011.
35	M	67	Yes	Mathis S, et al. 2011.
36	F	72	No	Mathis S, et al. 2011.
37	F	73	No	Koike H, et al. 2011.
38	M	48	Yes	Koike H, et al. 2011.
39	M	69	Yes	Koike H, et al. 2011.
40	M	56	Yes	Koike H, et al. 2011.
41	M	69	Yes	Koike H, et al. 2011.
42	M	76	Yes	Koike H, et al. 2011.
43	M	71	Yes	Koike H, et al. 2011.
44	F	61	Yes	Koike H, et al. 2011.
45	M	75	Yes	Koike H, et al. 2011.
46	M	69	Yes	Koike H, et al. 2011.

47	M	51	Yes	Koike H, et al. 2011.
48	M	73	Yes	Koike H, et al. 2011.
49	M	67	Yes	Koike H, et al. 2011.
50	M	54	Yes	Koike H, et al. 2011.
51	M	69	Yes	Koike H, et al. 2011.
52	M	60	Yes	Koike H, et al. 2012.
53	M	40	Yes	Koike H, et al. 2012.
54	M	28	Yes	Koike H, et al. 2012.
55	M	68	Yes	Meng LC, et al. 2015.
56	F	50	Yes	Meng LC, et al. 2015.
57	F	65	No	Meng LC, et al. 2015.
58	M	54	No	Bekircan-Kurt CE, et al. 2015.
59	M	54	Yes	Kollmer J, et al. 2017.
60	M	57	Yes	Kollmer J, et al. 2017.
61	M	70	Yes	Kollmer J, et al. 2017.
62	M	66	Yes	Kollmer J, et al. 2017.
63	M	74	Yes	Kollmer J, et al. 2017.
64	M	64	Yes	Du K, et al. 2021.
65	F	48	Yes	Du K, et al. 2021.
66	M	54	Yes	Du K, et al. 2021.
67	M	62	No	Du K, et al. 2021.
68	M	61	No	Du K, et al. 2021.
69	M	58	No	Du K, et al. 2021.
70	M	60	No	Du K, et al. 2021.

F, female; M, male.
